# Effects of Weight Loss on Echocardiographic Parameters 1 Year after Sleeve Gastrectomy

**Published:** 2019-07

**Authors:** Nasser Malekpour Alamdari, Fariba Bayat, Mahmood Bakhtiyari, Mehran Noori

**Affiliations:** 1 Modarres Hospital, Shahid Beheshti University of Medical Sciences, Tehran, Iran.; 2 Non-Communicable Diseases Research Center, Alborz University of Medical Sciences, Karaj, Iran.

**Keywords:** *Bariatric surgery*, *Echocardiography*, *Gastrectomy*

## Abstract

**Background:** Bariatric surgery is efficiently associated with the long-term resolution of obesity and its related morbidities. Not only can this surgical modality improve the metabolic profile, diastolic and systolic cardiac functions, and the clinical symptoms of heart failure or cardiomyopathy, but it can also reduce the atherosclerosis risk, ventricular mass, and pericardial fat thickness. The aim of the present study was to evaluate the effects of weight loss on echocardiographic parameters 1 year after sleeve gastrectomy.

**Methods:** This quasi-experimental study, conducted in Modarres Hospital, Tehran, Iran, from September 2016 to September 2017, recruited 101 patients. Those with morbid obesity (body mass index ≥40 kg/m^2^) had undergone surgery 1 year before the study commencement. All the patients underwent sleeve gastrectomy. The data of echocardiographic indices before and 1 year after surgery were recorded and analyzed.

**Results:** The mean age of the participants was 37.11±9.81 years. The majority of the study participants were female (n=77, 76.2%). During the first postoperative year, the mean weight loss was 43.82±14.53 kg. The mean systolic blood pressure was 137.96±19.60 mmHg and 123.37±9.60 mmHg before sleeve gastrectomy and 1 year afterward, respectively (P<0.001). The mean left ventricular size was 48.22±4.04 mm and 44.97±5.70 mm before sleeve gastrectomy and 1 year postoperatively, correspondingly (P<0.001). The mean pulmonary artery pressure was 28.88±5.25 mmHg and 24.10±4.78 mmHg before sleeve gastrectomy and 1 year after surgery, respectively (P<0.001). The mean left atrial size was 35.72±3.32 mm and 33.12±3.52 mm before sleeve gastrectomy and 1 year thereafter, correspondingly (P<0.001).

**Conclusion: **Weight loss resulting from sleeve gastrectomy may improve systolic blood pressure, left atrial and left ventricular size, and pulmonary artery pressure.

## Introduction

The prevalence of obesity has been dramatically increasing in both developed and developing countries over the last decades.^1^^, ^^2^ Obesity has been reported to have a prevalence rate of 12%–26% among the Iranian population.^3^^, ^^4^ As obesity is a unanimously accepted predisposing factor for many chronic diseases such as dyslipidemia, hypertension, diabetes mellitus, cardiovascular diseases, and sleep apnea, numerous cost-effective and sustainable treatments have been introduced and countless studies have been undertaken. 

Conservative intentional weight-loss strategies, including lifestyle modification and medication therapy, are generally considered inefficient in enduring weight management and diminishing the rate of cardiovascular events. So far, bariatric surgery has been the most effective intervention to obtain a sustained and significant weight loss in obese patients with a body mass index (BMI) >40 kg/m^2^. Bariatric surgery is efficiently associated with the long-term resolution of obesity and its related morbidities. Not only can this surgical modality enhance the metabolic profile, diastolic and systolic cardiac functions, and the clinical symptoms of heart failure or cardiomyopathy, but it can also diminish the atherosclerosis risk, ventricular mass, and pericardial fat thickness.^   5 ^

Sleeve gastrectomy is deemed the most effective method of bariatric surgery; it is a standard surgical method indicated in patients with severe morbid obesity.^6^^, ^^7^ A resolution to the co-morbidities allied to obesity, sleeve gastrectomy is easy to perform, results in minimal nutritional deficiencies, and is well tolerated by patients.^   8 ^

Previous studies have demonstrated that sleeve gastrectomy is capable of improving the cardiopulmonary function and its predisposing factors.^9^^-^^11^ Nevertheless, most of these studies have included follow-up periods of 3 and 6 months and failed to assess most cardiac function indices through modalities such as echocardiography. Therefore, the aim of our study was to evaluate the effects of weight loss on the cardiopulmonary function 12 months after sleeve gastrectomy.

## Methods

This before-and-after study conducted in Modarres Hospital, Tehran, Iran, from September 2016 to September 2017, recruited 130 patients. Those with morbid obesity (BMI ≥ 40 kg/m^2^) had undergone surgery 1 year prior to the commencement of the study. Based on the patients’ past medical history, physical examination results, and findings obtained from electrocardiography and echocardiography, those diagnosed with cardiac diseases (e.g., congenital heart diseases, valvular diseases, coronary artery diseases, regional wall motion abnormalities, heart failure, and dysrhythmias), untreated cardiopulmonary diseases, addiction, and psychological disorders, as well as those with poor image quality, were excluded from this study. Ultimately, the study population was composed of 101 patients.

The study protocol was approved by the Institutional Review Board at Shahid Beheshti University of Medical Sciences in accordance with the ethical guidelines of the Declaration of Helsinki. Written informed consent was also obtained from all the participants.

Demographic data; drug history; and past medical history of coronary vascular diseases, sleep apnea or snoring, hyper or hypotension, hypothyroidism, diabetes, and smoking were collected using a structured questionnaire through a face-to-face interview. Weight was measured, with subjects minimally clothed without shoes, using digital scales (Seca 707; range: 0.1–150 kg) and recorded to the nearest 100 g. Blood pressure was taken on the right arm at 2 intervals of 5 minutes after the patient rested for 15 minutes in a sitting position. A standardized mercury sphygmomanometer (calibrated by Iran’s Institute of Standards and Industrial Research) was utilized. Consequently, the mean of the measurements at the 2 intervals was calculated for analysis. Hypertension was defined as systolic blood pressure ≥140 mmHg and/ or diastolic blood pressure ≥90 mmHg or the current use of an antihypertensive medication based on the JNC7 (Joint National Committee 7). 

The participants were divided into 2 groups of current smokers, who smoked every day or occasionally, and nonsmokers, who had never smoked or had quit smoking. Type II diabetes was defined as fasting plasma glucose ≥7.0 mmol/L and/or 2-hour postprandial plasma glucose ≥11.1 mmol/L or being on antidiabetic medications. Subclinical hypothyroidism was defined as serum thyroid-stimulating hormone >5.06 mIU/L with a normal free T_4_ level. Overt hypothyroidism was defined as serum thyroid-stimulating hormone >5.06 mIU/L with free T_4_<0.91 ng/dL.

The BMI was calculated as weight (kg)/height×height (m^2^). BMI loss was calculated as preoperative BMI−current BMI. The study questionnaire was completed for all the patients 12 months after surgery.

Laparoscopic sleeve gastrectomy was performed by a single surgeon in our ward. The patients underwent surgery in the supine and reverse Trendelenburg position. Pneumoperitoneum was performed with the VisiPort under direct observation on the left side above the umbilicus. An inflation pressure of 12 mmHg was maintained. This procedure was performed with the use of 5 ports. First, the omentum was divided from the greater curvature of the stomach. Subsequently, with the use of a linear stapler (Echelon Flex, Johnson & Johnson), the stomach was resected about 6 cm proximal to the pylorus and on to the Hiss angle on a 32-F Bougie. The omentum was thereafter over-sewn continuously with absorbable 2-0 polydioxanone (PDS) sutures to reinforce the staple line. Next, a 16-F drain was placed along the staple line and externalized through the 5-mm port from the left flank. At the end of the procedure, the peritoneal cavity (above the stomach and under the diaphragm) and the bed of the spleen were irrigated with a bupivacaine 0.25% solution (30 cc) to reduce postoperative pain.^   12 ^

Echocardiographic studies were performed using a Sonos 7500 (Philips Ultrasound, Bothell, WA). Two-dimensional guided M-mode echocardiographic tracings in the parasternal long-axis view were performed to measure left atrial (LA) diameter and the parameters of left ventricular (LV) geometry. The mean pressure and the mean end-diastolic pressure in the pulmonary artery were directly assessed by measuring the peak and end-diastolic velocities of the pulmonary regurgitant jet using the following equations: a) diastolic pulmonary artery pressure (PAP) = pulmonary regurgitation end-diastolic pressure gradient + right atrial pressure and b) mean PAP = pulmonary regurgitation peak pressure gradient + right atrial pressure. Furthermore, diastolic dysfunction was assessed according to the recommendations of the American Society of Echocardiography and the European Association of Cardiovascular Imaging.

The demographic characteristics were presented as the mean (standard deviation [SD]) for the numerical variables and numbers (percentages) for the categorical measures. Departure from normality assumption was assessed using the Kolmogorov–Smirnov test. Differences in the descriptive baseline characteristics were explored using the Student independent *t*-test or the Mann–Whitney test (if necessary) between the independent groups. The differences between the cardiopulmonary parameters before and after sleeve gastrectomy were assessed using the paired *t*-test or the Wilcoxon signed-rank test. The independence of the 2 categorical variables was assessed by using the χ^2 ^test or the Fisher exact test. Additionally, the McNemar test was applied to compare the before-and-after paired nominal data. The association between weight loss during the follow-up and the cardiopulmonary function as the outcome was investigated through a generalized estimation equation (GEE) method. The GEE approach widely used an estimation method for the marginal (i.e., population-averaged) modeling of the repeated data. In brief, the GEE utilized the generalized linear model to estimate more efficient and unbiased regression parameters relative to ordinary least squares regression in part because they permit specification of a working correlation matrix that accounts for the form of the within-subject correlation of responses on the dependent variables of many different distributions, including normal, binomial, and Poisson. All the models were adjusted for age, gender, smoking status, hypothyroidism, sleep apnea, diabetes, and pulmonary diseases as time-varying confounders. All the analyses were performed separately for different obesity phenotypes using STATA statistical software (version 13 MP). Values <0.05 were considered statistically significant.

## Results

A total number of 101 morbidly obese patients were enrolled in this study. The demographic and clinical baseline characteristics of the participants are presented in Table 1. The mean±SD age of the participants was 37.11±9.81 years. The majority of the study participants were female (n=77, 76.2%). There were statistically significant differences between the female and male cases in the mean age and the distribution of cigarette smoking (P=0.027 and P<0.001, respectively).

During the first postoperative year, the mean weight loss was 43.82±14.53 kg. A history of sleep apnea was present in 51 (50.5%) patients before surgery, which decreased to 1 patient 1 year postoperatively (P<0.001). The McNemar test showed that the frequency of diabetes mellitus was not statistically decreased during the 1-year period after surgery (10% vs. 7.9%). 

The effects of weight loss on the study variables in the univariate analysis are demonstrated in Table 2. Weight loss resulting from sleeve gastrectomy was associated with an improvement in systolic blood pressure, LA size, LV size, and PAP.

The mean±SD systolic blood pressure was 137.96±19.60 mmHg and 123.37±9.60 mmHg before sleeve gastrectomy and 1 year after surgery, respectively (P<0.001). The mean (SD) LV size was 48.22±4.04 mm and 44.97±5.70 mm before sleeve gastrectomy and 1 year afterward, correspondingly (P<0.001). The mean±SD PAP was 28.88±5.25 mmHg and 24.10±4.78 mmHg before sleeve gastrectomy and 1 year thereafter, respectively (P<0.001). The mean±SD LA size was 35.72±3.32 mm and 33.12±3.52 mm before sleeve gastrectomy and 1 year postoperatively, correspondingly (P<0.001).

The results from the GEE analysis demonstrated a relationship between losing weight and the LV, PAP, and LA measures (Table 3). The results showed that a 1-kg decrease in body weight after sleeve surgery caused a reduction in the size of the LV by a coefficient of -0.01. Furthermore, in this model, the only significant factor that had a significant effect on LV size was the age of the patients during surgery inasmuch as an increase of 1 year in age increased the LV size by a coefficient of 0.099 (P=0.027).

Our results demonstrated that a reduction in weight per kg caused a statistically significant drop in PAP with a coefficient of 0.1. In addition, in this model, smoking (coefficient=2.59, P=0.006) and age (coefficient=0.11, P=0.003) affected PAP. The results also showed that a 1-kg weight loss caused a reduction in LA size by a factor of 0.05. The other variables that were entered into the model did not yield statistically significant results. Our findings showed no relationship between LV hypertrophy and diabetes mellitus and weight loss due to sleeve gastrectomy.

**Table 1:** Baseline and demographic characteristics of the female and male patients diagnosed with morbid obesity before surgery*

	Male (n=24)	Female (n=77)	P
BMI (km/m^2^)	44.41±4.44	45.9±4.7	0.861
PAP (mmHg)	28.20±5.40	31.21±4.10	0.007
LA size (mm)	35.22±3.26	37.31±3.20	0.008
LV size (mm)	4.72±4.23	50.10±2.71	0.002
LVH	1 (4.1)	18 (23.4)	0.032
Diastolic BP (mmHg)	79.32±13.12	84.71±6.72	0.570
Systolic BP (mmHg)	131.34±20.52	135.42 ±16.71	0.311
Weight (kg)	115.11±13.92	152.23±19.21	0.091
Smoking	11 (45.8)	6 (27.8)	<0.001
Hypothyroidism	3 (12.5)	19 (24.7)	0.262
Diabetes mellitus	1 (4.2)	5 (6.5)	0.462
Sleep apnea	18 (75)	33 (42.9)	0.063

BMI, Body mass index; PAP, Pulmonary artery pressure; LA, Left atrium; LV, Left ventricle; LVH, Left ventricular hypertrophy; BP, Blood pressure

* Data are presented as mean±SD or n (%).

**Table 2:** Univariate analysis of the differences between the cardiopulmonary parameters before and after sleeve gastrectomy*

	Before Sleeve Gastrectomy	After Sleeve Gastrectomy	P
Systolic blood pressure (mmHg)	137.96±19.60	123.37±9.60	<0.001
Diastolic blood pressure (mmHg)	81.61±13.55	79.40±4.85	0.231
Left ventricular size (mm)	48.22±4.04	44.97±5.70	<0.001
Pulmonary artery pressure (mmHg)	28.88±5.25	24.10±4.78	<0.001
Left atrial size (mm)	35.72±3.32	33.12±3.52	<0.001

* Data are presented as mean±SD.

**Table 3:** Effects of weight changes on the cardiopulmonary function indices following sleeve gastrectomy surgery

Outcome	Beta Coefficient (95% CI)	P
left ventricular size	-0.099 (-0.15 to -0.04)	<0.001
Pulmonary artery pressure	-0.1 (-0.15 to -0.05)	<0.001
Left atrial size	-0.051 (-0.09 to -0.01)	0.011
Left ventricular hypertrophy	1.0 (0.96 to 1.03)	0.883
Diastolic dysfunction	0.98 (0.95 to 1.03)	0.732

All models were adjusted for age, gender, smoking status, hypothyroidism disorders, diabetes disorders, and pulmonary disease as time-varying confounders

## Discussion

The results of the present study demonstrated the positive effects of weight loss on cardiac function by decreasing LV and LA size, as well as PAP. The increasing prevalence of obesity represents a major health burden worldwide and bariatric surgery, and in particular sleeve gastrectomy, has become an important pillar of treatment in severely obese patients.^13^^, ^^14^ Still, the absence of long-term outcomes in the studies hitherto conducted may raise concern over pathophysiological consequences beyond the weight loss obtained.^   15 ^

Impaired pulmonary and cardiac functions and indices are commonly observed in obese patients, even in those who are yet asymptomatic. Nevertheless, these changes are reversible following weight loss. Held and colleagues^   9 ^ showed a significant reduction in myocardial wall thickness after a 1-year weight-reduction program. Kokkinos and colleagues,^   10 ^ in a study of 37 morbidly obese patients, showed that all echocardiographic parameters improved significantly at 6 months in comparison with the baseline values. Benaiges and coworkers,^   11 ^ in a cohort study on 197 morbidly obese patients, reported a 68.1% reduction rate in hypertension 1 year after the surgical procedure. Previous studies have demonstrated that obesity is associated with an increase in wall thickness and chamber size.^   16 ^ Ayer et al.^17^ reported that the BMI was an independent determinant of LA size in their investigation. The findings from the abovementioned studies are in line with our study results. 

Laparoscopic sleeve gastrectomy was introduced as a restrictive procedure for obese patients, and it was initially described as a feasible first-stage operation in super-obese patients with BMI >60 kg/m^2^, as well as in high-risk patients, to a more complex definitive procedure.^   18 ^ In addition to its use in staged approaches in high-risk, high-BMI patients, laparoscopic sleeve gastrectomy is now commonly performed as a stand-alone bariatric operation for both high-risk and super-morbid-obese patients, as well as for patients with a lower BMI.^19^ Although it has been classified among the restrictive procedures, there is increasing evidence in the literature that laparoscopic sleeve gastrectomy acts with more than one mechanism. Studies have shown weight loss and diabetes resolution similar to Roux-en-Y gastric bypass.^   20 ^ Morbid obesity has become a worldwide concern because of the increase in the risk of diabetes, hypertension, heart disease, sleep apnea, and the other risk factors of cardiopulmonary disease.^   21 ^

Our result showed that the frequency of sleep apnea statistically decreased 1 year after surgery. The patients with diabetes mellitus after losing weight had a significant reduction in blood glucose levels. We also found that the frequency of diabetes mellitus was not statistically decreased during the 1-year period after surgery (10% vs. 7.9%). 

The findings of the current study should be confirmed by randomized controlled trials with a large sample size. Additionally, the measurements of body composition would be of interest, but test methods leading to valid data are a challenging issue in severe obesity. Moreover, it is already known that especially during the first months after sleeve gastrectomy, the marked decrease in body weight corresponds in part to fat-free mass. Therefore, patients should be re-evaluated after a longer period (i.e., 12–18 m) when weight loss has a higher impact on adiposity. These studies might rather focus on comparing different types of procedures such as Roux-en-Y gastric bypass, which is a simultaneously restrictive and malabsorptive operation.

## Conclusion

In our study population, weight loss resulting from sleeve gastrectomy improved high systolic blood pressure, PAP, and LA and LV size. 

## Notes:

***This paper should be cited as: ***Malekpour Alamdari N, Bayat F, Bakhtiyari M, Noori M. Effects of Weight Loss on Echocardiographic Parameters 1 Year after Sleeve Gastrectomy. J Teh Univ Heart Ctr 2019;14(3): 128-133.
